# A systematic comparison of intercultural and indigenous cultural dance education from a global perspective (2010–2024)

**DOI:** 10.3389/fpsyg.2024.1493457

**Published:** 2024-11-26

**Authors:** Weishan Liu, Hanbing Xue, Zi Yi Wang

**Affiliations:** ^1^Department of Early Childhood Education, Hunan Women's University, Changsha, China; ^2^School of Physics Science and Engineering, Tongji University, Shanghai, China; ^3^Department of Dance, Xinghai Conservatory of Music, Guangzhou, China

**Keywords:** cultural dance education, cultural identity, systematic literature review, global perspective, cross-cultural exchange

## Abstract

Cultural dance fosters social cohesion, emotional well-being, creative thinking, and cultural identity by conveying cultural values and social meanings. However, systematic comparative research on cross-cultural and indigenous cultural dance education remains limited. This study, adopting a global perspective, examines the current applications, core issues, and educational strategies in this field. Through a systematic literature review and grounded theory approach, the research scrutinizes studies conducted from 2010 to 2024. The results show that intercultural research primarily focuses on multicultural exchange, cultural integration of immigrant groups, and cultural acceptance among socioeconomically disadvantaged populations, highlighting the influence of intercultural adaptability and globalization. In contrast, indigenous cultural research emphasizes local culture and educational systems, with a particular focus on local cultural identity and educational reform. While both types of research reach a consensus on the importance of cultural transmission, diversity in teaching strategies, and the critical role of educators, significant differences remain in cultural identity and the design of educational content. Future research could concentrate on virtual dance education, innovation in indigenous cultures, the exploration of dance education’s role in mental health, and the deep integration of artificial intelligence into dance pedagogy. Furthermore, consideration of the potential impact of globalization and technological advancement on cultural identity and educational models will foster theoretical innovation and practical development in dance education.

## 1 Introduction

Dance, as a universal human activity, holds profound cultural and social significance. It serves as a medium for conveying cultural values (Hanna, 1987) and communicates through cultural codes (Bannerman, 2014). Individuals from diverse cultural backgrounds possess unique perceptions of dance space and bodily awareness (Grau, 2012). Thus, dance, with its multisensory cross-cultural experience, becomes a crucial medium for expressing cultural differences and social power dynamics (Desmond, 1993).

“Cultural dance,” defined as the dance of a specific cultural or ethnic group, often rooted in celebratory purposes—whether religious, secular, or social (Olvera, 2008)—has been increasingly utilized in dance education in recent years. In recent years, with the rapid advancement of globalization, cultural dance has gradually been incorporated into global education systems. Through multicultural exchange and interaction, it offers new possibilities for cross-cultural dialogue and understanding (Shapiro, 2008). It is not merely a form of physical expression but also a tool for enhancing clinical communication with patients (Darivemula et al., 2021). Moreover, it serves as a medium for conveying the values, beliefs, traditions, and histories of diverse groups, fostering social cohesion, emotional well-being, creative thinking, and cross-cultural understanding (Hanna, 1987).

However, the cultural homogenization brought about by globalization poses significant challenges to cultural dance education. Achieving an effective balance between multicultural integration and the tension of global cultural homogenization, while maximizing the potential of cultural dance education in promoting cross-cultural understanding and global cultural dialogue, has become a pressing issue.Despite numerous studies on cultural dance education in specific regions (Rowe, 2008; Gartzonika, 2013; Lewis and Ziady, 2021; Liu and Kalimyllin, 2024), systematic reviews, particularly comparative studies of cross-cultural and indigenous cultural dance education, remain scarce. This research gap limits our comprehensive understanding of dance education across different cultural contexts.

To address this gap, this study employs a systematic literature review to explore the similarities, differences, and developmental trends of intercultural and indigenous cultural dance education from a global perspective. By placing intercultural and indigenous cultural dance education within the same theoretical framework for systematic comparison, the research analyzes their educational strategies, core issues, and research perspectives across different cultural contexts. This analysis aims to reveal the impact of dance education on cultural identity, social values, and educational practices in varying cultural settings, providing theoretical support for future theoretical construction and educational practices in intercultural and indigenous cultural dance education. The specific research questions are as follows:

RQ1: What dynamic differences exist in the publication timeline, countries, institutions, and distribution of research subjects in intercultural and indigenous cultural dance education studies?

RQ2: What differences are observed in the core issues and application of theoretical frameworks in intercultural and indigenous cultural dance education research?

RQ3: What research perspectives exist on cross-cultural and indigenous cultural dance education? What similarities and differences are observed?

RQ4: What methods and strategies have been employed in cross-cultural and indigenous cultural dance education? How do they compare?

RQ5: What conclusions have been drawn from research on cross-cultural and indigenous cultural dance education? What are the similarities and differences?

## 2 Literature review

As early as 1963, researchers began to recognize the value of ethnic dance within cultural contexts (Kurath et al., 1963). Some scholars argue that all forms of dance should be viewed as ethnic dance within their specific cultural frameworks (Kealiinohomoku, 1970). The study of dance history and culture should be interwoven to fully comprehend the role of dance in various societies (Dils and Albright, 2001). Dance education should begin in early childhood to develop children’s awareness of movement and culture (Stinson, 1988), and it should also address power relations and social differences to foster critical thinking and social justice (Shapiro, 1998). With the growing academic focus on cultural dance education, research on both cross-cultural and indigenous cultural dance education has gained significant attention in the context of globalization (Bhabha, 2012).

### 2.1 Current research on cross-cultural dance education

Cross-cultural dance education encompasses a variety of approaches and perspectives. For example, multicultural dance education in the United States has suggested potential applications for Indonesian dance education (Masunah, 2008). Finnish teachers have adapted their teaching methods through cultural dances such as African dance, Oriental dance, and Flamenco (Siljamäki et al., 2010). In South Korea, a Laban expert expanded creative movement into public dance education, promoting “cultural democracy” (Hwang, 2013). Bharatanatyam, an Indian classical dance, has served as a tool for Asian Indian arts and cross-cultural literacy, influencing the cognition of Asian Indian children living in the West—particularly in terms of cultural identity and understanding (Iyengar, 2014). Dance companies in Australia and other regions have maintained emotional connections to tradition through organizational structures and adapted teaching principles (Mollenhauer, 2022). Studies have also addressed decolonization issues through cross-cultural dance education (Mejia, 2023) and enhanced cross-cultural reflective abilities (Mabingo et al., 2024). Existing research highlights the importance of cultural awareness, cultural democracy, ethical practices, and pedagogical adaptation in cross-cultural dance education, demonstrating its social value.

Meanwhile, research indicates adaptive challenges in cross-cultural anxiety screening (Wang et al., 2024; Gabunia et al., 2023), suggesting that cross-cultural dance education may face similar cultural adaptation issues in its promotion and application. This underscores the need for further investigation into its stability and practicality within diverse cultural contexts.

### 2.2 Current research on indigenous cultural dance education

Indigenous cultural dance education plays a crucial role in promoting cultural competence and inclusivity across various environments. For instance, Nannyonga-Tamusuza (2003) explored how the performance of the ba’a’kisimba dance redefined gender meanings. Banks (2007) investigated how Africans used dance to challenge colonial legacies, while Finkelstein (2022) showed that dance education can serve as a means of resistance and identity reconstruction. Phillips-Fein (2011) guided students to analyze dance within historical, social, and cultural contexts to develop critical thinking. Mabingo (2015) focuses on the analysis of policy documents, curricula, and practices within American dance education. Iacono and Brown (2016) explore dance as a living cultural heritage. Schupp (2019) reveals shifts in dance competition culture since the 1980s. Rosala and Budiman (2020) identify how traditional dance has been used to enhance students’ character in Indonesia. Liu and Kalimyllin (2024) investigate pathways for the transmission of Chinese culture through intangible cultural heritage dance. Existing research indicates that indigenous cultural dance education not only serves as a significant means of cultural expression but also fosters cultural diversity and social inclusivity.

Despite the growing body of research on intercultural and indigenous cultural dance education worldwide, and the rich theoretical contributions made by scholars in areas such as cultural heritage, multicultural education, and educational reform (Smith and Smith, 2013), several limitations still exist in the current studies. First, research on intercultural dance education tends to focus on cultural exchange and inclusivity within the context of globalization, but discussions on how to implement effective educational practices in different socioeconomic contexts are relatively insufficient. Second, while studies on indigenous cultural dance education emphasize the integration of cultural heritage and local educational systems, there is a lack of systematic exploration that combines modern teaching methods with the influences of globalization. Therefore, there remains considerable room for further exploration and deepening of the theoretical framework integration and practical application strategies in intercultural and indigenous cultural dance education research. These research limitations provide important entry points for subsequent investigations into the differences and commonalities between the two in terms of cultural understanding, social structural influences, and teaching strategies, while revealing the challenges and opportunities faced by the diversity of dance education and cultural heritage in the context of globalization.

## 3 Methodology and materials

### 3.1 Introduction to SLR methodology

This study employs the Systematic Literature Review (SLR) methodology (Xiao and Watson, 2019; Yang et al., 2022; Jensen and Bonde, 2018; Peng et al., 2024; Lin and Yu, 2024) to systematically and rigorously review and compare scholarly research on cultural dance education worldwide. The SLR approach ensures comprehensive literature coverage and reliability of research outcomes through its stringent procedures and transparent reporting requirements (Kushairi and Ahmi, 2021).

In practice, this study utilizes the standardized PRISMA screening process to select relevant literature (Page et al., 2021; Jin et al., 2024; Yu et al., 2024; Chen et al., 2024). An encoding system was established to collect objective data from the included literature (e.g., publication date, journal, research methods, and theoretical frameworks) and to extract textual coding information (e.g., core issues, authors’ perspectives, and research conclusions). The objective data were then subjected to quantitative statistical analysis and visual representation, while the textual coding information was analyzed using a combination of qualitative textual description and visualized charts.

### 3.2 Initial literature search

**Figure 1:**
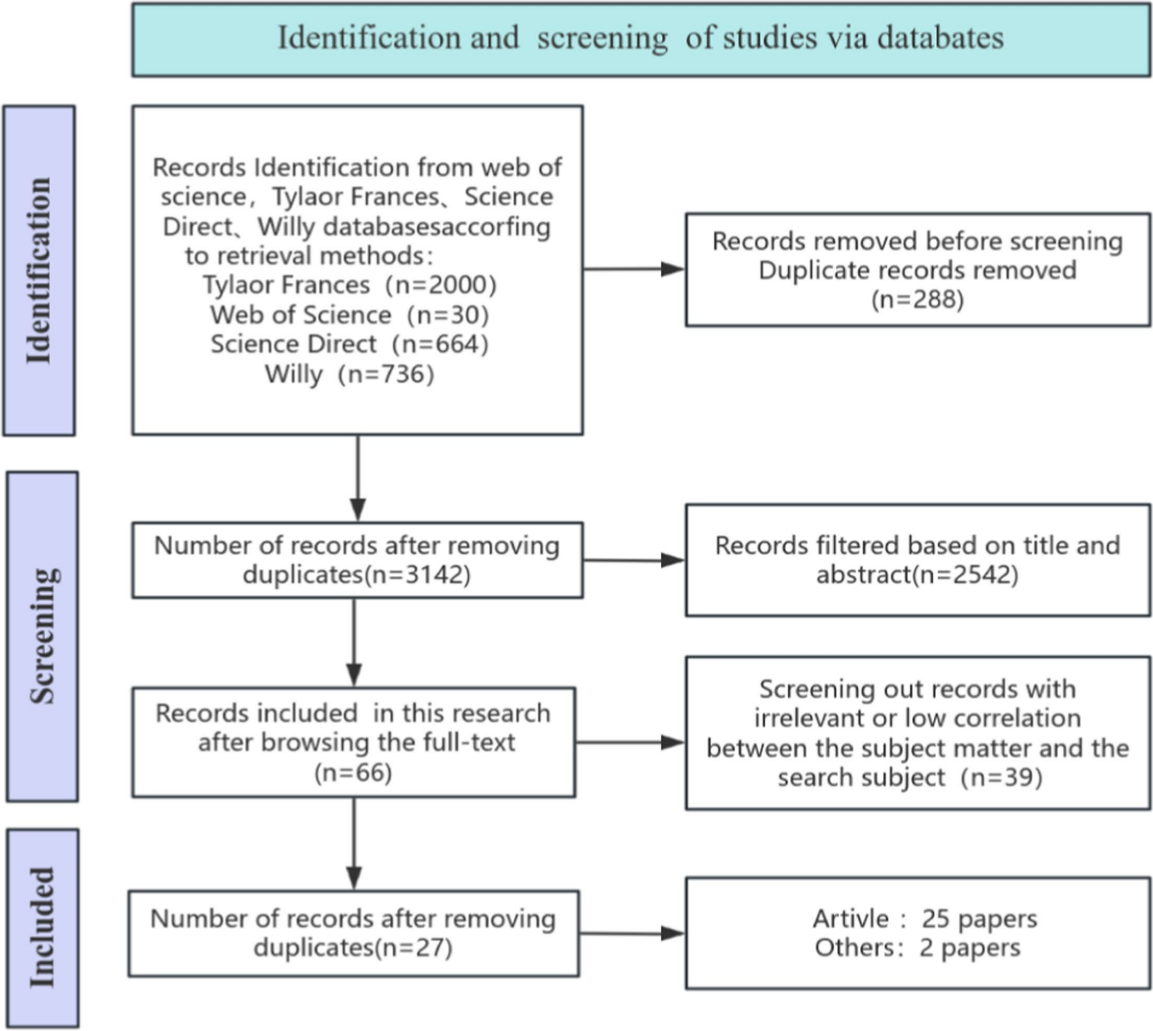
PRISMA Flowchart.

In alignment with the PRISMA guidelines (Page et al., 2021), we conducted a comprehensive literature search to gather the data required for this study. Four international English-language databases with the highest publication volume in this field were selected: Taylor & Francis, Web of Science, ScienceDirect, and Wiley. The inherent inclusion standards of these databases ensured that the selected literature generally met the quality requirements of the SLR. The search criteria were defined based on the study’s topics and objectives, and relevant keywords were determined by referencing related studies (Bhugra, 2005; Sanz-Camarero et al., 2023). For example, in the Web of Science database, the search string was: *TS = (“dance” AND “educati” AND “cultural identity” OR “Cultural Identity Construction” OR “Social Impact”)**. The search was conducted on April 28, 2024. The initial search across the five databases yielded a total of 3,142 documents after removing duplicates (the number of documents retrieved from each database is shown in Figure 1). Although five mainstream international English literature databases were selected to ensure the comprehensiveness of the literature data and the quality of the research, the random selection of databases may result in the exclusion of certain relevant studies, thereby introducing a risk of selection bias. Furthermore, considering that this study primarily relies on English databases for literature retrieval, there may be a lack of non-English literature, which could lead to an incomplete reflection of research findings on intercultural and indigenous cultural dance education within non-English contexts. These factors may pose challenges to the generalizability and comprehensiveness of the research conclusions, highlighting the need for future studies to further focus on the inclusion and analysis of multilingual literature.

### 3.3 Manual screening

During the screening process, we applied a set of clear criteria to select relevant literature. Inclusion criteria were as follows: (1) the literature must be peer-reviewed; (2) the research content must involve dance education within cross-cultural or indigenous cultural contexts; (3) the literature must explore topics related to cultural identity, educational reform, professional development, or social value; (4) the publication date must be after 2010; (5) the full text must be accessible online. Exclusion criteria included: (1) conference abstracts, review articles, books, and book reviews; (2) studies unrelated to dance education; (3) literature whose themes were not aligned with the research questions.

The manual screening was conducted in two stages. In the first stage, irrelevant literature was excluded based on titles and abstracts. This process took approximately 4 weeks, and two members of the research team were involved. After deduplication, 66 articles were retained from the five databases. In the second stage, a thorough reading of the full texts was conducted to pinpoint the literature required for this study. Three team members participated in this stage, which also took about 4 weeks, ultimately resulting in the retention of 27 articles. The complete literature screening process is illustrated in Figure 1. The citation information of the included literature is provided in Appendix A.

### 3.4 Analysis and coding

For the included literature, we systematically recorded critical information such as metadata, core issues, methodological strategies, authors’ perspectives, and research conclusions using standardized data extraction forms. Subsequently, a content analysis was conducted, in which the extracted data were coded and thematically categorized to identify and summarize the main characteristics and differences between cross-cultural and indigenous cultural dance education in areas such as cultural identity, identity construction, educational reform, teacher development, and social value representation. To ensure the objectivity and reliability of the analysis (Gaur and Kumar, 2018), a double-coding strategy was employed, where two researchers independently coded the data and resolved any discrepancies through discussion and consensus. Table 1 presents the coding framework used in this study.

**Table 1:** Coding Design Framework.

Research question	Category	Code
RQ1	Metadata	Publication date, publication venue, country of publication, research institutions
	Research subjects	Preschool children, primary school, middle school, high school, university, professional dancers, general public, specific groups (e.g., black, immigrant, low-income), teachers
	Theoretical framework	Types of theoretical frameworks included in the literature
RQ2	Core issues	Discussion and analysis of research topics and core themes in the included literature
RQ3	Research perspectives	Explanation of the core perspectives of authors in the included literature
RQ4	Methods and STRATEGIES	Introduction of teaching methods or strategies in the included literature
RQ5	Research conclusions	Explanation of research conclusions in the included literature

## 4 Performance analysis (RQ1)

### 4.1 Overview of performance indicators

#### 4.1.1 Publication time, venues, and volume

**Figure 2:**
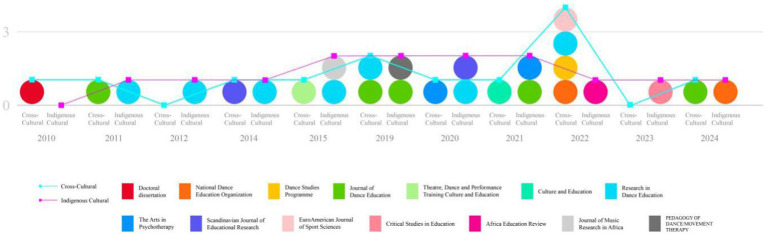
Articles published, the publication time and the comparison chart of publications.

To provide a clearer representation of the research trends, Figure 2 summarizes the specific data on publication timelines and journal distribution.

An analysis of the publication time and volume indicates a general upward trend in the number of studies on cross-cultural and indigenous cultural dance education from 2010 to 2024. Particularly after 2019, the volume of research in both areas has increased, reflecting the growing academic interest in cultural diversity and educational models in the context of globalization. Although research on indigenous cultural dance education slightly outnumbers cross-cultural studies overall, cross-cultural research has shown a steady increase in recent years, peaking in 2022 with four publications, far exceeding other years.

On the other hand, an analysis of publication venues reveals that research on both cross-cultural and indigenous cultural dance education has been featured across various academic platforms. Key journals such as the Journal of Dance Education and Research in Dance Education have served as important outlets for this type of research, providing a stable academic platform and reflecting the concentration and specialization of the field. Additionally, the National Dance Education Organization and the Dance Studies Program have offered significant supplementary perspectives, particularly in 2022, where cross-cultural studies were prominently featured. Indigenous cultural research, meanwhile, is distributed across a broader range of publications, including interdisciplinary journals such as Critical Studies in Education and Africa Education Review, highlighting the diversity and cross-disciplinary nature of the field.

Overall, the distribution of publication volume reflects the growing interest among scholars in cross-cultural exchange and educational models, while the distribution across publication venues indicates a broad academic exploration of cultural diversity and educational approaches, covering topics from professional dance education to sociocultural studies.

#### 4.1.2 National productivity and distribution of research institutions

**Figure 3:**
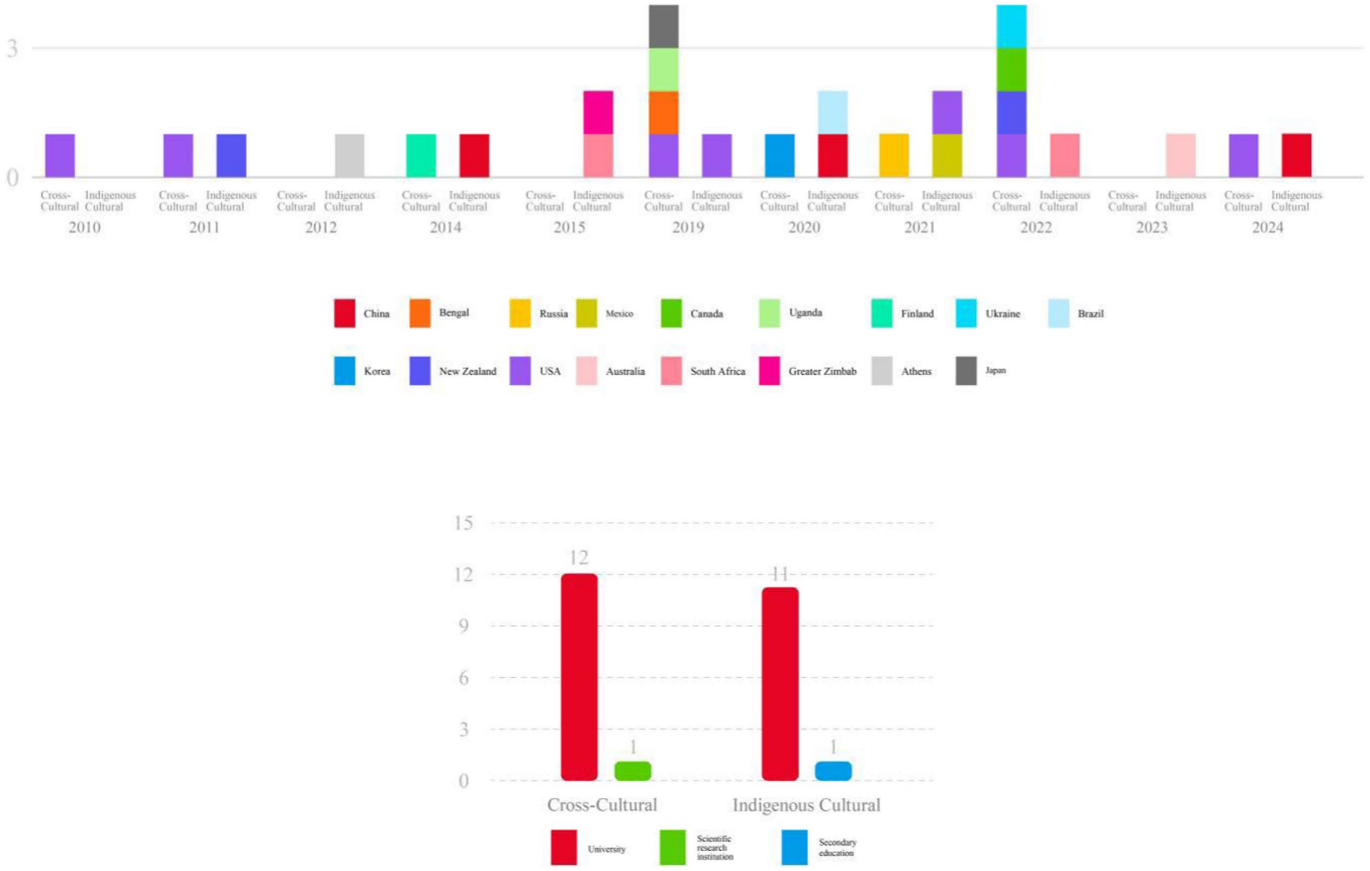
Comparison chart of national productivity and research institutions.

To more clearly illustrate the distribution of national productivity and research institutions, Figure 3 provides a visual comparison.

An analysis of national productivity and research institutions shows that cross-cultural dance education research involves nine countries, demonstrating broad international engagement and the characteristic of cross-cultural exchange. Indigenous cultural dance education research involves 10 countries, indicating the depth and extensive coverage of indigenous cultural studies in specific regions. Additionally, cross-cultural dance education research spans 12 universities and one research institution, highlighting the professionalism and concentration of academic research. In contrast, indigenous cultural studies also involve 12 universities, with the addition of one secondary education institution and two studies without specified institutions, suggesting that indigenous cultural research is conducted not only in universities but also across different levels of educational institutions.

In summary, cross-cultural dance education research reflects global academic collaboration and the exploration of diverse cultural backgrounds, showcasing international cooperation. In contrast, indigenous cultural research demonstrates strong academic support and in-depth local research within specific countries and regions. The distribution of core productivity in both fields reflects the differences in research focus and institutional support.

#### 4.1.3 Characteristics of research participants

To detail the differences in research participants between cross-cultural and indigenous cultural dance education, Table 2 provides a comparative summary of the various research participants.

**Table 2:** Research participants in cross-cultural vs. indigenous cultural dance education.

Analysis type	Categories of research participants
Cross-cultural	Adults aged 5–70, low-income Black youth aged 4–15, immigrants and students from different countries, non-professional dancers, students from primary, secondary, and tertiary education, White teachers and female teachers, professional dancers and dance artists
Indigenous cultural	Students from primary, secondary, and tertiary education, dance teachers, the general public, U.S. educators, Greek traditional dance practitioners, professional dancers, adults, Indigenous learners

The research team conducted a comparative analysis of the research participants involved in cross-cultural and indigenous cultural dance education studies, The results are as shown in Table 3.

**Table 3:** Comparative analysis of research participants.

Analysis type	Cross-cultural	Indigenous cultural
Diversity of research participants	Covers various age groups, socioeconomic backgrounds, races, and professional backgrounds, with a particular focus on immigrants, students from different countries, and low-income Black youth	Focuses on students and teachers within the educational system and specific cultural groups (e.g., Greek traditional dance practitioners, Indigenous learners)
Social background	Emphasizes diversity in social backgrounds, such as immigrants, low-income youth, and students from different countries	Focuses on groups within specific cultural and educational systems, such as U.S. educators, Greek traditional dance practitioners, and Indigenous learners
Educational stages	Spans all age groups and educational stages, from children to adults	Covers students across different educational stages
Balance of professional and non-professional	Balances research on professional and non-professional dancers, exploring the accessibility of dance education and professional development	Also addresses professional dancers, but places greater emphasis on students and teachers within the educational system

These findings reveal that cross-cultural dance education research features greater diversity and breadth in its participants, with a strong emphasis on multicultural backgrounds and issues of social equity. In contrast, indigenous cultural dance education research is more focused on groups within specific cultural and educational systems, highlighting the importance of cultural heritage and educational practices. This comparison provides direction for future research, allowing researchers to select appropriate subjects based on specific needs to achieve more comprehensive and in-depth study results.

#### 4.1.4 Theoretical frameworks

Table 4 summarizes the theoretical frameworks used in cross-cultural and indigenous cultural dance education research, highlighting the theoretical support within different research contexts.

**Table 4:** Theoretical frameworks in cross-cultural vs. indigenous cultural dance education.

Type of literature	Theoretical frameworks
Cross-cultural	Practical Theology Theory (A1); Ethnocentrism Theory (A3); Bourdieu’s Theory (A5); Communication Theory (A6); Critical Clan Theory (A8); Vertical Osmosis Concept and Constructivist Theory (A10); Cultural Studies (A12)
Indigenous cultural	Funnell’s Cross-Cultural Practice (B1); Respect Design Theory (B2); Cultural Interface Theory (B3); Sociology (B5); Kinetic Nationalism (B7); Adult Learning Theory (B8); Sociocultural Theory (B9); Bourdieu’s Theory (B10); Constructivism (B12); Embodiment Theory (B14)

* References corresponding to A1, A3, etc., can be found in Appendix B; references corresponding to B1, B2, etc., can be found in Appendix C.

The research team conducted a further analysis of the theoretical frameworks applied in cross-cultural and indigenous cultural dance education studies, as presented in Tables 5, 6.

**Table 5:** Commonality analysis of theoretical framework applications.

Commonality category	Cross-cultural	Indigenous cultural
Cultural understanding and respect	Practical Theology Theory (A1), Ethnocentrism Theory (A3), Cultural Studies (A12), etc., emphasize understanding and respect for dance across different cultural backgrounds	Respect Design Theory (B2), Cultural Interface Theory, Kinetic Nationalism (B3), etc., highlight the importance of Indigenous culture and ethnic identity(B7)
Social structure and power dynamics	Bourdieu’s and Foucault’s frameworks analyze power structures and social stratification in dance training (A5, A8)	Bourdieu’s Theory reveals the influence of teachers on student training and competition (B10), while sociological perspectives analyze the impact of social structures on dance education (B5)
Teaching strategies and the role of teachers	The Vertical Osmosis Concept and Constructivist Theory emphasize the role of teachers in constructing cultural identity (A10)	Adult Learning Theory (B8) and Bourdieu’s Theory emphasize the role of teachers in curriculum planning and student training (B10)

**Table 6:** Differentiation analysis of theoretical framework applications.

Differentiation category	Cross-cultural	Indigenous cultural
Cultural exchange and interaction	Communication Theory emphasizes the role of traditional dance in cultural exchange, promoting cross-cultural understanding	Cultural Interface Theory emphasizes the convergence and interaction of different cultures within education
Religious belief	Practical Theology Theory reveals the influence of religion on dance through studies of Hinduism	/
Cultural identity	Critical Clan Theory redefines Black dance aesthetics, emphasizing cultural identity in the era of new nationalism	Kinetic Nationalism emphasizes the expression and reinforcement of ethnic identity through dance movements
Sociocultural environment	Cultural Studies emphasize the application of teaching philosophies by dance teachers in cross-cultural contexts	Sociocultural Theory clarifies the dynamics of teaching and cultural processes within a fluid social and cultural organizational environment

The analysis reveals that, in terms of cultural understanding, both cross-cultural and indigenous frameworks value cultural understanding and respect, emphasizing cultural diversity and inclusivity in dance education. Regarding social structure and power dynamics, both explore the role of these elements in dance education, uncovering the impact of power relations on the educational process. In teaching strategies and the role of teachers, both highlight the importance of teachers in dance education, particularly in curriculum design and implementation.

The differentiation analysis shows that, in the realm of cultural exchange and interaction, cross-cultural dance education focuses more on exchange and interaction between cultures, while indigenous cultural dance education concentrates on interactions at cultural intersections. In terms of religious belief, cross-cultural dance education gives greater attention to the influence of religion and belief systems on dance, a topic less explored in indigenous cultural dance education. Concerning cultural identity, cross-cultural dance education emphasizes identity in the context of new nationalism, whereas indigenous cultural dance education focuses on the relationship between dance movements and ethnic identity. In the sociocultural environment, cross-cultural dance education emphasizes the role of teachers within cross-cultural contexts, while indigenous cultural dance education addresses the impact of dynamic sociocultural environments on the educational process.

### 4.2 Thematic analysis (RQ2)

#### 4.2.1 Open coding

To gain a deeper understanding of the core issues in dance education within cross-cultural and indigenous cultural contexts, we conducted a systematic analysis of the selected literature. Tables 7 and 8 summarize the primary research themes and contributions of both in areas such as cultural identity, educational reform, professional development, and social value.

**Table 7:** Core issues in cross-cultural dance education research.

Research theme	Research theme
Cultural heritage and identity	Several studies explore how dance education promotes cultural heritage and identity, such as the teaching of classical South Indian dance in the U.S. (A1); the role of traditional ethnic dance in intercultural communication among elementary school students (A5)
Cross-cultural exchange and integration	Many studies focus on the role of dance education in cross-cultural exchange, such as the impact of contact improvisation courses on multi-ethnic identity (A3); the cross-cultural significance of Ugandan dance (A9)
Teaching methods and educational reform	Several topics discuss specific teaching methods and the necessity of educational reform, such as the pedagogical method of integrating recreational culture (A7); teaching experiences of East Asian dance/movement therapy educators (A8); the introduction of specific writing practices in cross-cultural dance (A11)
Social context and power structures	Some studies examine the influence of social context and power structures on dance education, such as power dynamics in Rihab’s choreography choices (A4); cultural transfer in Canadian diaspora sports dance studios (A6)
Criticality and reflection	Critical research and reflection are important research directions, such as exploring racialized orientations using critical phenomenology (A2); revealing cross-cultural significance through the reflections of student performers (A10)
Impact of globalization	The impact of globalization on dance education is also a significant topic, such as the role of art education systems in globalization (A13); exploring the teaching philosophies of Finnish cross-cultural dance teachers (A12)

* References corresponding to A1–A13 can be found in Appendix D.

**Table 8:** Core issues in indigenous cultural dance education research.

Research theme	Conceptual meaning
Cultural heritage and identity	Some studies focus on cultural identity and heritage, such as evaluating respondents’ perceptions of Chinese dance (B1), reconstructing localized contexts of Indigenous culture (B2), reconstructing traditional dance teaching (B4), introducing educational regulations for Greek dance (B5), achieving ethnic cultural goals (B6), assessing Russian university students’ attention to Chinese dance (B7), and developing students’ cultural identity (B14)
Educational reform and inclusivity	Several topics address reform and inclusivity strategies within the educational system, such as the professional development of physical education teachers in traditional dance teaching (B8), the impact of community art projects on disadvantaged children (B9), and a case study of professional development programs for dance education in New Zealand elementary schools (B12)
Professional development and identity	Some studies focus on the identity and professional development of professional dancers, such as identity crises among South African dance practitioners (B3), professional development experiences of physical education teachers (B8), the impact of dance competitions on students’ identity formation (B10), and the development of students’ professional identity through DMT (B14)
Social value and impact	Some topics address the social value reflected in dance education, such as how free dance performance reflects the relationship between tradition and innovation (B11) and dance students’ self-perception of the health benefits of West African dance (B13)

* References corresponding to B1–B14 can be found in Appendix E.

Overall, the core issues in cross-cultural dance education encompass various countries and cultural backgrounds, reflecting the diversity and breadth of cross-cultural dance education. Additionally, these issues involve multiple disciplines, including culture, education, sociology, and theology, highlighting the interdisciplinary nature of the research. Many studies not only explore theoretical questions but also focus on teaching methods and practice, such as discussions of specific teaching methods and suggestions for curriculum reform.

On the other hand, the broad scope and profound impact of research on indigenous cultural dance education emphasize the multiple roles of dance in culture, education, profession, and society.

#### 4.2.2 Axial coding

Building on this, we further analyzed the commonalities and differences of these themes through axial coding, as shown in Tables 9, 10, aiming to reveal how cross-cultural and indigenous cultural dance education advances these core issues in similar and different contexts.

**Table 9:** Comparative analysis of core issues—commonalities.

Commonality category	Cross-cultural	Indigenous cultural
Cultural identity and heritage	Investigates the role of dance education in promoting cultural heritage and identity, such as the teaching of classical South Indian dance in the U.S. (A1) and the role of ethnic dance in intercultural communication (A5)	Focuses on cultural identity and heritage, including perceptions of Chinese dance (B1), the reconstruction of Indigenous culture (B2), and the reconstruction of traditional dance teaching (B4)
Educational reform and teaching methods	Discusses the necessity of specific teaching methods and educational reform, such as integrating recreational culture into pedagogy (A7) and the introduction of writing practices in cross-cultural dance (A11)	Emphasizes reform and inclusivity strategies within the education system, such as the professional development of physical education teachers (B8) and the impact of community art projects on disadvantaged children (B9)
Criticality and reflection	Employs critical and reflective approaches, such as critical phenomenology (A2) and revealing cross-cultural significance through student performers’ reflections (A10)	Not directly mentioned but embedded in critical reflection within education reform and professional development
Professional development and identity	Involves the professional development of teachers and educators’ teaching experiences (A8)	Explicitly explores the identity and professional development of professional dancers, such as identity crises among dance practitioners (B3) and the development of professional identity through DMT (B14)

**Table 10:** Comparative analysis of core issues—differences.

Differentiation category	Cross-cultural	Indigenous cultural
Geographical and cultural scope	Covers various countries and cultural backgrounds, focusing on cross-cultural exchange and educational reform within a globalized context	Primarily focuses on the culture and educational practices of a single country or region, with greater emphasis on local cultural identity and heritage
Research perspective and focus	Focuses on exploring cross-cultural exchange and the impact of globalization	Emphasizes local cultural identity, educational reform, and professional development, with attention to specific teaching practices and social value
Teaching methods and practices	Emphasizes diverse teaching methods and cross-cultural integration	Focuses on specific teaching experiences and local educational reform

Both types of research emphasize cultural identity and heritage, educational reform and the development of teaching methods, criticality and reflection, as well as the exploration of professional development and identity.

In summary, both cross-cultural and indigenous cultural dance education share similarities in their core issues, such as an emphasis on cultural heritage, educational reform, and social impact. However, significant differences also exist. Cross-cultural research highlights cultural exchange and critical analysis within a globalized context, emphasizing cross-cultural exchange and diverse teaching methods. On the other hand, indigenous cultural research focuses on the cultural identity, educational practices, and professional development of specific countries, with an emphasis on local cultural heritage and the improvement of teaching experiences. These comparisons not only reveal the multidimensional impact of dance education in different cultural and social contexts but also provide rich perspectives and directions for future research.

### 4.3 Analysis of research perspectives (RQ3)

#### 4.3.1 Open coding

In our in-depth analysis of research on intercultural and indigenous cultural dance education, we utilized open coding to summarize the core perspectives of each body of work. Tables 11, 12 reveal unique insights into key themes such as cultural exchange, pedagogical methods, identity formation, social function, and the integration of innovation and tradition.

**Table 11:** Core perspectives in intercultural dance education research.

Research perspective category	Conceptual implications
Intercultural exchange and understanding	Any form of isolation and segregation leads to mutual misunderstanding. Traditional songs and dances are suitable for fostering positive intercultural, religious, and ethnic exchanges and should be incorporated into teaching (A6). The process of teaching and learning non-Western cultural dances plays a significant role in promoting intercultural learning in higher education (A10). Practicing intercultural dance may be a way to enhance cultural understanding (A12).
Cultural adaptation and pedagogical reform	Pedagogical cultural adaptation is the process through which dance educators modify and negotiate teaching techniques and methods to suit new cultural environments and communities (A3). Cultivating comprehensive cultural dance literacy, moving beyond a Eurocentric monocultural perspective (A8). Introducing Dance/Movement Therapy (DMT) in non-Western cultural contexts requires adapting to students’ learning styles while integrating local cultural perspectives on psychotherapy (A9). When writing and dance practices are conducted in a conscious and interconnected manner, they can facilitate student learning in both areas (A11).
Race and identity formation	Without acknowledging racialized discourse, dance curricula will continue to perpetuate narratives of racial inferiority, leading students to experience racialized “otherness” (A2). Dance education for Indian classical dancers abroad must include improvisation training to enhance dance proficiency and foster the development of multi-group racial identity (A4).
Social function of dance education	Dance conveys social concepts such as community, authority, and respect (A1). Soviet socialist body ideology influenced the Soviet-Canadian second-generation immigrant ballroom dancers and their teachers, shaping the direction of ballroom dancing in North America and Europe (A7).
Integration of innovation and tradition	The protection of choreography must be pursued globally, with modern dance forms receiving equal emphasis as traditional forms (A13). ·Dance arts education will continue to perpetuate both traditional and modern dance forms. Despite traditional practitioners questioning the authenticity of contemporary styles, their appeal makes them a vital entry point for new dancers (A13).

*For the original references corresponding to the perspectives in intercultural research, please see Appendix F.

**Table 12:** Core perspectives in indigenous cultural dance education research.

Research perspective category	Conceptual implications
Cultural heritage and national identity	The role of Chinese dance in preserving cultural heritage in the digital age, particularly concerning collective and national identity (B1). The positive impact of students acquiring additional cultural knowledge on learning specific ethnic dances, including the understanding of traditions, culture, and attitudes (B6). · Culturally-oriented dance has potential positive effects on the physical and mental health of professional dancers and communities, particularly regarding the health benefits of traditional West African dance (B12).
Pedagogical methods and practices	Broadening mainstream pedagogical approaches by valuing the voices of elders and Indigenous Knowledge Systems (IKS), incorporating cultural practices such as dance into teaching methods, and emphasizing the value of lifelong learning (B2). The philosophy of physical training should include creativity related to metacognitive qualities, such as self-reflection, self-efficacy, and resilience (B3). Adjusting dance pedagogy through anthropological theories, emphasizing the necessity of creating open and trusting environments for successful teaching (B7). Through the positive practice of DMT in the classroom, DMT educators can share experiences with the broader world of educational theory, promoting interdisciplinary practice (B14). Community arts projects and equitable teaching frameworks are essential (B9). The application of anthropological theory allows dance pedagogy to be adapted according to the teacher’s objectives and purposes (B8)
Cultural reproduction and creativity	Dance education practices impact post-colonial countries’ social justice pedagogies, aiding in the restoration of African cultural identity (B4). · The process of socialization may limit participants’ creativity and agency, influencing the reproduction of dance culture and shaping individual and collective identities (B10).
Sociocultural role of dance	Sekper, rooted in local animistic religious practices, has limited information available to outsiders (B5). Frevo dance, as a dance of resistance, challenges the limitations of “tradition” and “authenticity,” serving both as cultural heritage and a form of personal artistic expression (B11). Dance plays a significant role in the development of multicultural education and culturally responsive pedagogy, helping children explore and express culture (B12). Culturally-oriented dance has potential positive effects on the physical and mental health of professional dancers and students in higher education and community dance programs (B13).

*For the original references corresponding to the perspectives in indigenous cultural research, please see Appendix G.

In summary, the analysis of the core perspectives in intercultural dance education highlights the multifaceted role of dance in intercultural exchange, cultural adaptation, racial identity, social functions, and the integration of innovation and tradition. These studies collectively emphasize dance as not merely an art form but a crucial tool for cultural transmission, social norm education, and identity construction. Through dance education, understanding and integration between different cultures can be enhanced, thereby promoting equity and inclusivity in education while also preserving and disseminating dance heritage globally.

In conclusion, research on indigenous cultural dance education underscores the multifaceted roles of dance in cultural heritage preservation, national identity, pedagogical innovation, cultural reproduction, and the sociocultural role of dance. The studies demonstrate that dance not only plays a vital role in personal health, cultural understanding, and pedagogical practices but also has profound implications for social justice and cultural restoration.

#### 4.3.2 Axial coding

When analyzing the authors’ perspectives on cross-cultural and indigenous cultural dance education, we can identify certain commonalities and differences. Tables 13, 14 provide a comparative analysis of these commonalities and differences, helping to reveal similarities and distinctions between the two in areas such as cultural exchange and identity, teaching methods, identity formation, social functions, and the integration of innovation with tradition.

**Table 13:** Comparative analysis of commonalities between intercultural and indigenous cultural perspectives.

Commonality category	Intercultural	Indigenous
Cultural exchange and identity	Emphasizes the importance of intercultural exchange, viewing traditional dance as an effective medium for fostering intercultural understanding (A5, A9, A11)	Emphasizes that learning dance within specific cultural contexts deepens understanding and identification with that culture, aiding students in exploring and expressing themselves in a multicultural environment (B1, B6, B12)
Teaching methods	Highlights the need for teachers to adapt their methods in intercultural contexts (A3, A7, A8, A10)	Stresses the importance of integrating indigenous knowledge systems and creating a trusting environment, focusing on the application of local culture and traditions in teaching (B2, B3, B7, B8, B9, B14)
Identity formation	Addresses the negative impact of racialized discourse on students’ identity, emphasizing the role of dance education in promoting multiethnic identity formation (A2, A4)	Focuses on the restoration of cultural identity in post-colonial nations, examining the impact of dance cultural reproduction on identity formation (B4, B10)
Social function	Emphasizes the social functions of dance in intercultural contexts, such as the transmission of community authority and respect (A1, A6)	Focuses on the social roles of dance within specific cultures, including religious practices and expressions of resistance to tradition (B5, B11, B12, B13)
Integration of innovation and tradition	Advocates for the balanced preservation of both modern and traditional dance forms on a global scale (A12, A13)	Focuses on the impact of traditional dance on community and individual health, reflecting an emphasis on culturally-oriented approaches (B4, B10, B12, B13)

**Table 14:** Comparative analysis of differences between intercultural and indigenous cultural perspectives.

Difference category	Intercultural	Indigenous
Cultural exchange and understanding	Focuses on intercultural adaptation and exchange	Emphasizes the protection of cultural heritage and national identity
Teaching methods	Highlights cultural adaptation and integration within multicultural contexts	Focuses on broadening mainstream pedagogical approaches and valuing indigenous knowledge systems
Race and identity formation	Concerns with the impact of racialized discourse on students’ identity	Examines the role of cultural reproduction in shaping individual and collective identity
Social function	Focuses on the global social impact of dance	Concentrates on the social role of dance within local communities
Integration of innovation and tradition	Stresses the equal importance of modern and traditional dance	Focuses on the positive impact of culturally-oriented dance on community and individual health

Intercultural and indigenous cultural dance education share common ground in cultural exchange, teaching methods, identity formation, social function, and the fusion of innovation with tradition. However, they also exhibit significant differences. Intercultural research emphasizes adaptation and diversified teaching methods within a global context, while indigenous cultural research centers on the preservation of local traditions and the improvement of specific teaching practices. These comparisons not only illuminate the multidimensional impact of dance education across different cultural and social contexts but also offer valuable perspectives and directions for future research.

In summary, the analysis of differences between intercultural and indigenous cultural dance education in areas such as cultural exchange, teaching methods, identity formation, social function, and the integration of innovation with tradition reveals their differing priorities. Intercultural research is inclined towards adaptation within a globalized context, while indigenous cultural research places greater emphasis on the preservation of local traditions and teaching practices. This comparison not only highlights theoretical and practical differences but also provides valuable insights for the diversified development of dance education.

### 4.4 Methodological strategies (RQ4)

#### 4.4.1 Open coding

When analyzing the methodological strategies of cross-cultural and indigenous cultural dance education, we extracted key strategies through open coding. Tables 15, 16 aim to reveal how these strategies enhance students’ cultural understanding and identity, as well as the global development of dance education.

**Table 15:** Methodological strategies in intercultural dance education.

Strategy category	Conceptual connotation
Social interaction and cultural transmission	Employ social interaction, teaching, and performance practices to convey new forms of knowledge (A1) Use storytelling and music to activate specific movements, enhancing teacher-student relationships (A3) Create a safe space through humor to foster mutual sharing among students (A9)
Stratified and group-based teaching	Implement stratified group teaching to assign students to appropriate roles in dance productions (A1) Utilize methods such as group discussions and whole-class sharing (A9, A11)
Teacher training and trauma-informed dance therapy	Establish culturally relevant teacher training to help educators understand and convey cultural knowledge (A2) Employ trauma-informed dance therapy to aid students in healing from trauma and promote mental well-being (A2)
Authoritarian and autocratic teaching methods	Utilize authoritarian teaching methods, placing teachers in positions of power for rigorous training (A7)
Critical and reflective teaching	Provide theoretical approaches to critical inquiry, emphasizing student independence and creativity (A8, A9)
Cultural integration and tradition	Apply a unified teaching approach that integrates traditional costumes and props to reveal contextual knowledge of dance (A10) Reflect multiculturalism through the inclusion of Flamenco culture, West African dance, and more (A12) Incorporate local culture through practices such as “Tai Chi” and “meditation” (A9) Teachers use Eastern Indian folk dance to shape students’ ideologies (A5)
Inclusivity and equity	Establish scholarships to support student learning and development, with a focus on diversity and inclusivity in curriculum design (A1)

* A4, A6, and A13 do not provide descriptions of methodological strategies. The original coding information for intercultural strategies can be found in Appendix H.

**Table 16:** Methodological strategies in indigenous cultural dance education.

Strategy category	Conceptual connotation
Cultural collaboration and exchange	·Emphasizes cultural collaboration and experiential exchange in teaching strategies, promoting cultural transmission and understanding through concerts, dance classes, special events (B1), carnivals, and other activities (B11)
Inclusivity and collaboration	·The teaching environment emphasizes inclusivity and collaboration, utilizing indigenous methods such as Yarning to encourage participants to share and learn in an inclusive, collaborative setting (B2) ·Mixed teaching and cooperation with peers and families emphasize collaboration over competition, reflecting inclusivity and collaboration (B9)
Cultural responsiveness and critical reflection	·Teaching methods focus on cultural responsiveness, developing curriculum content and pedagogical knowledge through interaction, problem-solving, observation, feedback, and critical reflection (B12)
Care and support	·Teachers’ care and support for students are reflected in the teaching process, emphasizing the combination of effort and caution through posture adjustments and attention to students’ physical needs (B9)
Practice and performance	·Emphasizes motivating students through competition and performance, enhancing their skills and confidence (B6, B10, B11)

* B3, B4, B5, B7, B8, B13, and B14 do not provide descriptions of methodological strategies. The original coding information for indigenous cultural strategies can be found in Appendix I.

The teaching methods and strategies in intercultural dance education exhibit diversity and breadth. Through social interaction, cultural transmission, critical inquiry, stratified teaching, and diverse teaching methods, these strategies promote students’ holistic development. Not only do they facilitate understanding and identification with different cultures, but they also provide effective teaching strategies and theoretical foundations for the globalization of dance education.

The teaching methods and strategies in indigenous cultural dance education also demonstrate diversity and breadth. Through cultural collaboration and exchange, inclusivity and collaboration, cultural responsiveness and reflection, care and support, and practice and performance, these strategies promote students’ cultural identity, critical thinking, and personalized development.

#### 4.4.2 Axial coding

Building on the open coding, we further conducted axial coding to deeply analyze the commonalities and differences in the strategies of intercultural and indigenous cultural dance education. Tables 17, 18 aim to reveal the commonalities and distinct characteristics of these strategies across different cultural contexts, as well as how they collectively influence the practical outcomes of dance education and cultural transmission.

**Table 17:** Comparative analysis of commonalities in methodological strategies between intercultural and indigenous cultural perspectives.

Commonality category	Intercultural	Indigenous
Cultural collaboration and exchange	Promotes cultural transmission and understanding through social interaction, storytelling, music, and multicultural forms such as Flamenco and West African dance (A1, A3, A12)	Advances cultural dissemination and exchange through activities such as concerts, dance classes, and carnivals (B1, B11)
Inclusivity and collaboration	Fosters inclusivity and equity through diverse teaching methods, including stratified teaching and group discussions (A1, A9)	Uses indigenous methods like Yarning and mixed teaching to encourage collaboration over competition, with a focus on inclusivity in education (B2, B9)
Critical reflection	Emphasizes student independence and creativity through critical inquiry and reflective teaching (A8, A9)	Develops curriculum content and pedagogical knowledge through interaction, problem-solving, feedback, and critical reflection (B12)
Care and support	Promotes students’ mental health through trauma-informed dance therapy and supportive teaching practices (A2)	Stresses the importance of teachers’ care for students’ physical and psychological well-being, supporting their overall development (B9)
Practice and performance	Integrates performance activities and multiculturalism into education (A1, A10, A12)	Cultivates students’ practical skills and confidence through competitions and performances (B6, B10, B11)

**Table 18:** Comparative analysis of differences in methodological strategies between intercultural and indigenous cultural perspectives.

Difference category	Intercultural	Indigenous
Cultural integration and collaborative exchange	Emphasizes cultural integration and tradition through unified teaching methods, combining traditional costumes and props, and the integration of different cultures, such as the ideological shaping of Indian folk dance	Focuses on the preservation and display of local culture, showcasing cultural characteristics through activities like carnivals
Teacher training and trauma-informed dance therapy	Includes culturally relevant teacher training and trauma-informed dance therapy design	/
Authoritarian and autocratic teaching methods	The application of authoritarian and autocratic teaching methods places teachers in positions of authority for rigorous training	/
Social interaction and cultural transmission	Emphasizes social interaction and interaction strategies that convey new knowledge through teaching and performance practices	Focuses on promoting understanding and identification through cultural collaboration and exchange

Research on intercultural and indigenous cultural dance education reveals significant commonalities in methodological strategies. Both approaches promote cultural understanding through various forms of cultural exchange, emphasize inclusivity and collaboration, value critical reflection and students’ mental health, and focus on practice and performance activities. These strategies collectively enhance the inclusivity and effectiveness of cultural dance education.

The comparative analysis of differences reveals significant contrasts in the practical implementation and theoretical application of teaching strategies between intercultural and indigenous cultural dance education. Intercultural dance education focuses more on cultural integration, diversity, and diverse teaching styles, while indigenous cultural dance education emphasizes the preservation and display of local culture, inclusivity, and collaboration. These differences reflect the distinct cultural backgrounds and educational objectives of each approach.

### 4.5 Analysis of research conclusions (RQ5)

#### 4.5.1 Open coding

In summarizing the research on intercultural and indigenous cultural dance education, open coding was utilized to delve deeply into various research conclusions. Tables 19, 20 present the research findings on how dance education within cross-cultural and localized contexts promotes cultural adaptation, teaching reform, and teacher development.

**Table 19:** Research conclusions in intercultural dance education.

Research conclusion category	Conceptual content
Cultural adaptation and identity	Cultural Adaptation: Enhancing students’ adaptability in cross-cultural environments by adjusting teaching strategies and integrating diverse cultural elements (A3, A7, A10). · Cultural Identity: Fostering students’ sense of cultural identity through dance education, while promoting understanding and respect for other cultures, thereby strengthening cultural identity (A3, A5, A6).
Diverse teaching methods	Multifaceted Approaches: Promoting comprehensive student development by integrating language and music, modifying movements, modern choreography, and improvisation to innovate cultural works (A3, A4, A11, A13). · Integrated Teaching: Combining dance with other disciplines such as practical theology and figurative writing to enhance students’ overall abilities and interdisciplinary thinking (A1, A5).
Teacher roles and capabilities	Teacher’s Role: Teachers serve not only as knowledge transmitters but also as cultural mediators and facilitators of adaptability in the classroom (A12). · Professional Development: Teachers need to actively address students’ racial differences and develop new professional identities to adapt to a multicultural context (A2, A9).
Cultural inclusivity and equity	· EEP Dance Teaching Model: Overcoming traditional cultural biases by enhancing cultural inclusion and educational equity (A8).
Student agency	Learning Agency: Through reflection and inquiry, students actively construct cross-cultural meaning, enhancing their cultural understanding and learning experience (A10). · Learning Experience: Through diverse learning approaches, students deepened their understanding and mastery of dance and its underlying cultural connotations (A1, A4).

* The original coding information for the intercultural research conclusions can be found in Appendix J.

**Table 20:** Research conclusions in indigenous cultural dance education.

Research conclusion category	Conceptual content
Cultural transmission and identity	Complexity of Chinese Dance Culture: Students’ insufficient understanding of Chinese dance culture may hinder the global integration of Chinese culture (B1, B7). Transmission of Indigenous Knowledge: Jagun’s indigenous knowledge, passed down by elders to the younger generation, promotes cultural identity and strengthens community ties (B2). The Role of Ethnic Dance in Greek Education: Ethnic dance promotes cultural homogeneity and national identity (B6). Cultural Significance of Ballet Folklórico: Assists Mexican-American students in developing identity and pride (B9).
Teaching methods	Limitations of Western Pedagogies: Western-centric pedagogies reinforce implicit norms and stereotypes, suggesting the need for self-reflection and resilience training (B3). Teaching Methods of Guerreiros do Passo: Preserves tradition while encouraging student innovation, fostering cultural debate (B11). Culturally Responsive Strategies in Dance Teaching: Strengthens connections between teachers and students, among students, and between students and dance (B12). Embodied Self-Reflection in DMT Students: Enhances awareness of cultural, professional, and bodily selves through experiential and embodied learning (B14).
Teacher capabilities	Inadequate Teacher Capabilities: Teachers’ limited ability to demonstrate and execute traditional dance calls for improvements in teacher training and pedagogical methods (B4, B5). Challenges for Japanese Physical Education Teachers: Japanese PE teachers face challenges in creating broad-based instructional plans that respect the regional characteristics of traditional dance and address inappropriate teaching formats (B8).
Social function and impact	Impact of Dance Competitions: Intensive training for dance competitions helps shape individual traits, reproduce cultural preferences, and build team spirit among participants (B10). Impact of West African Dance: West African dance contributes to dancers’ physical, psychological, and social well-being, connecting culture and community, although time and financial constraints pose barriers to participation (B13).

*No research conclusion is provided for B7; the original coding information for the indigenous cultural research conclusions can be found in Appendix K.

The key findings of cross-cultural dance education research emphasize cultural adaptation and identity in the context of globalization, diverse teaching strategies, teacher roles and competencies, cultural inclusion and equity, as well as the emphasis on student agency in learning. By integrating tradition and innovation, these studies not only transmit technical skills but also develop rich cultural meanings, promoting cross-cultural understanding and exchange. These conclusions provide valuable insights and guidance for the development of intercultural dance education.

The research conclusions on indigenous cultural dance education primarily emphasize cultural transmission, teacher roles, cultural diversity, and community involvement. These studies highlight the multifaceted functions of dance as a tool for cultural education, serving not only as a vehicle for cultural preservation but also as a means of promoting psychological well-being and social connectivity. The research also identifies challenges within dance education and proposes corresponding improvements to enhance the overall effectiveness of dance education. Overall, these studies provide rich perspectives and practical guidance for indigenous cultural dance education, underscoring the unique value of dance within the educational system.

#### 4.5.2 Axial coding

In further axial coding, we conducted a comparative analysis of the commonalities and differences in existing research findings. Tables 21, 22 clearly illustrate the different methodological approaches, social purposes, and interconnections between cross-cultural and indigenous cultural dance education in practice.

**Table 21:** Comparative analysis of commonalities in cross-cultural vs. indigenous cultural research conclusions.

Commonality category	Cross-cultural	Indigenous culture
Cultural transmission and identity	Global Adaptation: Utilizing diverse cultural elements to help students adapt to a global environment (A3, A7, A10) Cross-Cultural Understanding: Enhancing recognition and respect for one’s own and others’ cultures (A3, A5, A6)	Indigenous Transmission: Emphasizing the cultural role of indigenous knowledge and folk dance (B2, B6) Cultural Identity: Promoting identification with one’s own culture (B1, B7, B9)
Diverse teaching methods	Integrated Approaches: Using various methods and interdisciplinary integration to promote development (A3, A4, A11, A13) Interdisciplinary: Integrating multiple fields to enhance overall abilities (A1, A5)	Strategic Response: Focusing on specific teaching strategies and cultural responsiveness (B3, B11) Teaching Adaptation: Addressing cultural differences through teaching strategies (B12, B14)
Teacher roles and competencies	Multiple Roles: Teachers as transmitters of knowledge and facilitators of cultural adaptation (A2, A9, A12) Professional Development: Emphasizing adaptation to cultural differences (A2, A9)	Skills Challenges: Discussing teacher training and skill enhancement (B4, B5) Regional Adaptation: Adjusting teaching methods to accommodate regional cultural characteristics (B9)

**Table 22:** Comparative analysis of differences in cross-cultural vs. indigenous cultural research conclusions.

Difference category	Cross-cultural	Indigenous culture
Cultural scope of teaching content	Involves multicultural integration and a global perspective	Focuses on cultural transmission specific to certain countries or regions
Social purpose of dance education	Emphasizes cross-cultural understanding and identity formation	Discusses the role of dance education in social and political spheres
Practical innovations in dance education	Encourages free improvisation and interdisciplinary dance teaching to foster student creativity and understanding	Emphasizes implementing cultural response strategies and experiential learning in dance education to strengthen student connections with dance

Both cross-cultural and indigenous cultural dance education center around the core objectives of cultural transmission and identity. They play a crucial role in the preservation of national cultures and the enhancement of cultural identity. Diverse teaching methods are emphasized in both contexts: cross-cultural research highlights integrated approaches, while indigenous cultural research focuses on specific strategies. Teacher roles and competencies are also crucial, with teachers needing to fulfill multiple functions in facilitating cultural adaptation and professional development. These commonalities indicate that the effectiveness of dance education relies on diverse teaching methods, multifaceted teacher roles, and sensitivity to cultural differences.

In summary, a comprehensive review of existing research conclusions in cross-cultural and indigenous cultural dance education reveals fundamental differences in cultural content selection. In terms of social and political purposes, dance education exhibits distinct social functions across different cultural and geographical contexts. In practical innovations, cross-cultural and indigenous cultural dance education each reflect unique characteristics in educational practices and innovative methods, providing clear perspectives for future researchers to understand teaching designs and objectives within different educational contexts.

## 5 Conclusion

Research into cross-cultural and indigenous cultural dance education is growing globally, with a notable increase in cross-cultural studies in recent years, reflecting a heightened emphasis on multiculturalism and cross-cultural exchange. Cross-cultural research encompasses a wide range of socio-economic contexts, with particular emphasis on immigrant and low-income groups, demonstrating a high degree of cultural inclusivity. This aligns with the discussions by several scholars (Outevsky, 2024; Amie, 2022) on the role of cultural education in promoting social equity and inclusive development. In contrast, indigenous cultural research focuses more on specific regions and educational groups, emphasizing cultural preservation and innovative development (Wiggins, 2015; Liu, 2020). Although there are similarities in the theoretical frameworks used to explain the impact of culture and social structures, notable differences exist in terms of cultural interaction, religious beliefs, and cultural identity. These differences provide a solid theoretical foundation for exploring cultural diversity in global dance education (Q1).

In terms of core issues, cross-cultural dance education focuses on cultural exchange within the context of globalization, emphasizing cultural identity and educational reform and innovation. Studies show that cross-cultural education fosters the understanding and acceptance of diverse cultures (Rahman, 2015; Ko, 2020). In contrast, indigenous cultural dance education focuses on the preservation and development of local culture, with a particular emphasis on fostering students’ cultural identity and sense of belonging, while also addressing the professional development of educators and dancers (Sato et al., 2021; Day et al., 2024). Additionally, it highlights the preservation of local culture and its integration with practical teaching methods, aligning with previous studies (Johnson et al., 2022) that underscore the significance of cultural identity in the context of globalization. Verall, cross-cultural research prioritizes exploring cultural interaction and educational innovation from a global perspective, whereas indigenous research places greater emphasis on local cultural preservation and the continuation of traditions (Q2).

In terms of research perspectives, scholars of cross-cultural dance education not only focus on the expression of dance as an art form but also emphasize its role in cultural adaptation, dissemination, and innovative integration within a global context. Dance is considered a significant cultural practice tool, serving multiple functions such as cultural transmission, social education, and identity construction. In contrast, researchers in indigenous cultural dance education place greater importance on the preservation and application of cultural heritage, recognizing the vital role of dance in promoting personal health, enhancing cultural understanding, national identity, and cultural reproduction (Q3).

In terms of teaching strategies, cross-cultural dance education emphasizes cultural integration and diversity, promoting students’ comprehensive development through various methods such as social interaction, cultural transmission, and critical inquiry. Indigenous cultural dance education, on the other hand, focuses on cultural collaboration and practice, fostering students’ cultural identity and critical thinking through inclusion, cooperation, and performance. Both approaches value cultural understanding and students’ mental health, but cross-cultural education concentrates on integration and innovation within a globalized context, while indigenous education highlights the preservation and transmission of local culture, reflecting their respective educational goals and cultural backgrounds (Q4).

In the research conclusions, both approaches emphasize the significance of dance in cultural transmission, the diversity of teaching methods, and the role of educators. However, cross-cultural dance education focuses on cross-cultural integration, while indigenous cultural dance education concentrates on the preservation and display of local culture, revealing differences in educational objectives and practical innovations (Q5). This finding aligns with the discussions in existing research (Banks, 2007; Hwang, 2013; Finkelstein, 2022), further emphasizing the central role of cultural diversity in education.

Based on the above research findings, this study proposes a “Cross-Cultural-Indigenous Cultural Symbiosis Model.” This model integrates the core commonalities and differences of cross-cultural and indigenous cultural dance education within a single analytical framework to reveal the dynamic interactions and symbiotic relationships between the two cultural systems in the context of globalization. The model consists of three core dimensions: Cultural Coexistence, which emphasizes the complementarity of cross-cultural and indigenous cultural education systems in educational objectives and cultural transmission, thereby enhancing mutual cultural understanding and social inclusivity (Kymlicka, 1995); Cultural Fusion, focusing on the integration strategies of cross-cultural dance education within multicultural teaching methods and a global perspective to promote mutual understanding among diverse cultures (Banks, 2006; Dervin, 2016); and Cultural Uniqueness, which highlights the protection and innovation of the unique values of indigenous cultural dance education in cultural identity, beliefs, and educational practices, fostering self-identity and innovative development of indigenous culture in the context of globalization (Homi, 1994; Hall, 1990). We believe that the “Cross-Cultural-Indigenous Cultural Symbiosis Model” not only helps to explain the core dynamic relationships within the research on cross-cultural and indigenous cultural dance education but also provides theoretical guidance for future studies. This is particularly relevant in addressing the challenge of effectively balancing cultural fusion with the preservation of indigenous culture in the context of rapid globalization and digitalization.

## 6 Future prospects

### 6.1 Cross-cultural dance education

#### 6.1.1 Digital transformation and virtual dance education

Virtual reality technology offers the advantage of providing immersive experiences, breaking down geographical and cultural boundaries, and showcasing unique value in cross-cultural dance education. Future research could further explore the development of virtual dance classrooms and analyze their effectiveness in promoting cultural understanding and identity. For example, designing virtual dance classrooms to offer cross-cultural dance courses and conducting effectiveness assessments. Additionally, the application of digital tools such as online collaboration platforms and artificial intelligence deserves in-depth study, particularly regarding their potential and actual impact in enhancing cross-cultural communication, supporting personalized learning, and advancing global educational cooperation.

#### 6.1.2 Local innovation in the context of globalization

With the acceleration of globalization, maintaining the uniqueness of local dance in dance education has become increasingly important. Future research could focus on the impact of globalization on local art creation, exploring the representation of indigenous dance in cross-cultural dance festivals and art exhibitions, and its contribution to global cultural exchange. Additionally, it is equally important to analyze the role of cross-cultural dance festivals and exhibitions in promoting global cultural exchange and artistic innovation. For example, observing the representation of indigenous dance at international dance festivals while also assessing how local innovations gain recognition within the global cultural landscape can provide valuable insights.

#### 6.1.3 Psychological and sociological innovations in cultural dance education

The application of dance in psychotherapy is gaining increasing attention due to its ability to help people from diverse cultural backgrounds address psychological issues through body language. Future research could examine the therapeutic effects and practical applications of dance in different cultural environments. Additionally, it is worth further exploring how social networks and online communities influence cultural dance education, particularly how these platforms foster interaction and cultural understanding among learners in local cultural contexts. These studies will help uncover new opportunities in dance education and cultural exchange.

#### 6.1.4 Application of artificial intelligence in cross-cultural education

Artificial intelligence technology offers broad possibilities for innovation in cross-cultural dance education. Future research could focus on how to utilize AI to design personalized learning pathways, assist in teaching, provide timely feedback, and enhance students’ learning outcomes and experiences. Furthermore, studying the application of AI-generated dance works in teaching and their potential impact on students’ creativity and cultural understanding could open new development directions for cultural dance education.

#### 6.1.5 Future cultural identities and dance

With the rapid development of information technology, the connotations and expressions of cultural identity are undergoing profound changes. Future research could delve deeper into how dance education adapts to these changes, especially how dance teaching can make the transmission and expression of cultural identity more diverse in a digital environment. Additionally, exploring the role of dance education in cross-cultural communication, and how it helps students better understand and express diverse cultural identities, can contribute to the shaping of emerging cultural identities in a global context.

### 6.2 Local cultural dance education

#### 6.2.1 Cross-cultural adaptation of local dance

In the context of globalization, effectively protecting and promoting indigenous dance is a topic of significant research value. Future studies should focus on the performance and adaptation of indigenous dance on the international stage, analyzing its unique role in fostering international cultural exchange and cooperation. Additionally, research should further examine how these strategies enhance the recognition of indigenous cultures, providing a better understanding of the role and significance of local cultures in the globalization process. For example, designing an international dance festival that invites artists from various countries to perform indigenous dances together can enhance cultural identity and strengthen mutual understanding.

#### 6.2.2 Cultural mixing and innovation in education

Future research should investigate how to effectively integrate global cultural elements into indigenous dance education to develop innovative hybrid cultural curricula. This involves analyzing specific methods for incorporating global cultural elements into traditional dance teaching and evaluating their impact on students’ cultural awareness and learning experiences. Such studies will offer valuable insights for future teaching innovations. For example, creating a series of cross-cultural dance workshops that allow students to experience various dance styles from different cultural backgrounds while learning traditional dances.

#### 6.2.3 Modern transformation of traditional dance

The modernization of traditional dance is an unavoidable topic in the era of globalization. Future research should explore how to blend traditional dance with modern dance forms to promote the preservation and development of traditional dance. Specifically, research could investigate the integration of traditional dance with digital arts, such as digital performances and virtual stages, analyzing the impact of these new technologies on the development of traditional dance and seeking new avenues for its modernization. For example, an augmented reality application could be developed that allows audiences to view digital performances of traditional dances on their mobile devices, thereby attracting the interest of the younger generation in traditional dance.

#### 6.2.4 Community-driven dance education innovations

Communities play a crucial role in the implementation of cultural dance education and therefore warrant greater attention. Future research should actively explore how communities participate in and influence cultural dance education, evaluating community-led dance projects and educational models. Additionally, it should address the key role of local artists in cultural preservation and the development of local art, analyzing their impact on community dance education and their contributions to cultural preservation. For example, organizing community dance performances to encourage local artists and residents to participate together can strengthen the sense of identity among community members regarding their indigenous culture.

#### 6.2.5 Deepening equity and diversity in education

In the context of globalization, educational equity remains an unavoidable contemporary issue. Future research should delve into the implementation pathways for diversity and equity strategies in indigenous dance education, focusing on how to effectively address these issues to ensure fair and inclusive educational practices. Evaluating the impact of these strategies on student learning outcomes and exploring how to achieve educational equity in multicultural environments are essential. By systematically analyzing practices related to diversity and equity, this research will provide a basis for the scientific formulation of educational policies and promote the development of fairness and diversity in the educational field. For example, conducting dance course assessments tailored to students from diverse backgrounds can ensure the diversity of course content and teaching methods, promoting equal participation for all students.
