# Supplementary material for: A systematic comparison of intercultural and indigenous cultural dance education from a global perspective (2010–2024)
Source: Front Psychol. 2024 Nov 26;15:1493457. doi: 10.3389/fpsyg.2024.1493457 (PMC11628298; doi:10.3389/fpsyg.2024.1493457)
Supplement: Supplementary file 1 [file Data_Sheet_1.docx]

Supplementary Tables

**Table 1. **Coding Design Framework****

| **Research Question** | **Category** | **Code** |
| --- | --- | --- |
| RQ1 | Metadata | Publication Date, Publication Venue, Country of Publication, Research Institutions |
|  | Research Subjects | Preschool Children, Primary School, Middle School, High School, University, Professional Dancers, General Public, Specific Groups (e.g., Black, Immigrant, Low-Income), Teachers |
|  | Theoretical Framework | Types of Theoretical Frameworks Included in the Literature |
| RQ2 | Core Issues | Discussion and Analysis of Research Topics and Core Themes in the Included Literature |
| RQ3 | Research Perspectives | Explanation of the Core Perspectives of Authors in the Included Literature |
| RQ4 | Methods and Strategies | Introduction of Teaching Methods or Strategies in the Included Literature |
| RQ5 | Research Conclusions | Explanation of Research Conclusions in the Included Literature |

**Table 2. **Research Subjects in Cross-Cultural vs. Indigenous Cultural Dance Education****

| Analysis Type | Categories of Research Subjects |
| --- | --- |
| Cross-Cultural | Adults aged 5-70, low-income Black youth aged 4-15, immigrants and students from different countries, non-professional dancers, students from primary, secondary, and tertiary education, White teachers and female teachers, professional dancers and dance artists |
| Indigenous Cultural | Students from primary, secondary, and tertiary education, dance teachers, the general public, U.S. educators, Greek traditional dance practitioners, professional dancers, adults, Indigenous learners |

**Table 3. Comparative Analysis of Research Subjects**

| **Analysis Type** | **Cross-Cultural** | **Indigenous Cultural** |
| --- | --- | --- |
| Diversity of Subjects | Covers various age groups, socioeconomic backgrounds, races, and professional backgrounds, with a particular focus on immigrants, students from different countries, and low-income Black youth | Focuses on students and teachers within the educational system and specific cultural groups (e.g., Greek traditional dance practitioners, Indigenous learners) |
| Social Background | Emphasizes diversity in social backgrounds, such as immigrants, low-income youth, and students from different countries | Focuses on groups within specific cultural and educational systems, such as U.S. educators, Greek traditional dance practitioners, and Indigenous learners |
| Educational Stages | Spans all age groups and educational stages, from children to adults | Covers students across different educational stages |
| Balance of Professional and Non-Professional | Balances research on professional and non-professional dancers, exploring the accessibility of dance education and professional development | Also addresses professional dancers, but places greater emphasis on students and teachers within the educational system |

**Table 4. Theoretical Frameworks in Cross-Cultural vs. Indigenous Cultural Dance Education**

| **Type of Literature** | **Theoretical Frameworks** |
| --- | --- |
| Cross-Cultural | Practical Theology Theory (A1); Ethnocentrism Theory (A3); Bourdieu’s Theory (A5); Communication Theory (A6); Critical Clan Theory (A8); Vertical Osmosis Concept and Constructivist Theory (A10); Cultural Studies (A12) |
| Indigenous Cultural | Funnell’s Cross-Cultural Practice (B1); Respect Design Theory (B2); Cultural Interface Theory (B3); Sociology (B5); Kinetic Nationalism (B7); Adult Learning Theory (B8); Sociocultural Theory (B9); Bourdieu’s Theory (B10); Constructivism (B12); Embodiment Theory (B14) |

*Note: References corresponding to A1, A3, etc., can be found in Appendix B; references corresponding to B1, B2, etc., can be found in Appendix C.

**Table 5. Commonality Analysis of Theoretical Framework Applications**

| **Commonality Category** | **Cross-Cultural** | **Indigenous Cultural** |
| --- | --- | --- |
| Cultural Understanding and Respect | Practical Theology Theory (A1), Ethnocentrism Theory (A3), Cultural Studies (A12), etc., emphasize understanding and respect for dance across different cultural backgrounds | Respect Design Theory (B2), Cultural Interface Theory, Kinetic Nationalism (B3), etc., highlight the importance of Indigenous culture and ethnic identity(B7) |
| Social Structure and Power Dynamics | Bourdieu’s and Foucault’s frameworks analyze power structures and social stratification in dance training (A5, A8) | Bourdieu’s Theory reveals the influence of teachers on student training and competition (B10), while sociological perspectives analyze the impact of social structures on dance education (B5) |
| Teaching Strategies and the Role of Teachers | The Vertical Osmosis Concept and Constructivist Theory emphasize the role of teachers in constructing cultural identity (A10) | Adult Learning Theory (B8) and Bourdieu’s Theory emphasize the role of teachers in curriculum planning and student training (B10) |

**Table 6. Differentiation Analysis of Theoretical Framework Applications**

| **Differentiation Category** | **Cross-Cultural** | **Indigenous Cultural** |
| --- | --- | --- |
| Cultural Exchange and Interaction | Communication Theory emphasizes the role of traditional dance in cultural exchange, promoting cross-cultural understanding | Cultural Interface Theory emphasizes the convergence and interaction of different cultures within education |
| Religious Belief | Practical Theology Theory reveals the influence of religion on dance through studies of Hinduism | / |
| Cultural Identity | Critical Clan Theory redefines Black dance aesthetics, emphasizing cultural identity in the era of new nationalism | Kinetic Nationalism emphasizes the expression and reinforcement of ethnic identity through dance movements |
| Sociocultural Environment | Cultural Studies emphasize the application of teaching philosophies by dance teachers in cross-cultural contexts | Sociocultural Theory clarifies the dynamics of teaching and cultural processes within a fluid social and cultural organizational environment |

**Table 7. Core Issues in Cross-Cultural Dance Education Research**

| **Research Theme** | **Research Theme** |
| --- | --- |
| Cultural Heritage and Identity | Several studies explore how dance education promotes cultural heritage and identity, such as the teaching of classical South Indian dance in the U.S. (A1); the role of traditional ethnic dance in intercultural communication among elementary school students (A5) |
| Cross-Cultural Exchange and Integration | Many studies focus on the role of dance education in cross-cultural exchange, such as the impact of contact improvisation courses on multi-ethnic identity (A3); the cross-cultural significance of Ugandan dance (A9) |
| Teaching Methods and Educational Reform | Several topics discuss specific teaching methods and the necessity of educational reform, such as the pedagogical method of integrating recreational culture (A7); teaching experiences of East Asian dance/movement therapy educators (A8); the introduction of specific writing practices in cross-cultural dance (A11) |
| Social Context and Power Structures | Some studies examine the influence of social context and power structures on dance education, such as power dynamics in Rihab’s choreography choices (A4); cultural transfer in Canadian diaspora sports dance studios (A6) |
| Criticality and Reflection | Critical research and reflection are important research directions, such as exploring racialized orientations using critical phenomenology (A2); revealing cross-cultural significance through the reflections of student performers (A10) |
| Impact of Globalization | The impact of globalization on dance education is also a significant topic, such as the role of art education systems in globalization (A13); exploring the teaching philosophies of Finnish cross-cultural dance teachers (A12) |

**Table 8. Core Issues in Indigenous Cultural Dance Education Research**

| **Research Theme** | **Conceptual Meaning** |
| --- | --- |
| Cultural Heritage and Identity | Some studies focus on cultural identity and heritage, such as evaluating respondents' perceptions of Chinese dance (B1), reconstructing localized contexts of Indigenous culture (B2), reconstructing traditional dance teaching (B4), introducing educational regulations for Greek dance (B5), achieving ethnic cultural goals (B6), assessing Russian university students' attention to Chinese dance (B7), and developing students' cultural identity (B14) |
| Educational Reform and Inclusivity | Several topics address reform and inclusivity strategies within the educational system, such as the professional development of physical education teachers in traditional dance teaching (B8), the impact of community art projects on disadvantaged children (B9), and a case study of professional development programs for dance education in New Zealand elementary schools (B12) |
| Professional Development and Identity | Some studies focus on the identity and professional development of professional dancers, such as identity crises among South African dance practitioners (B3), professional development experiences of physical education teachers (B8), the impact of dance competitions on students’ identity formation (B10), and the development of students' professional identity through DMT (B14) |
| Social Value and Impact | Some topics address the social value reflected in dance education, such as how free dance performance reflects the relationship between tradition and innovation (B11) and dance students' self-perception of the health benefits of West African dance (B13) |

*Note: References corresponding to B1-B14 can be found in Appendix E.

**Table 9. Comparative Analysis of Core Issues—Commonalities**

| **Commonality Category** | **Cross-Cultural** | **Indigenous Cultural** |
| --- | --- | --- |
| Cultural Identity and Heritage | Investigates the role of dance education in promoting cultural heritage and identity, such as the teaching of classical South Indian dance in the U.S. (A1) and the role of ethnic dance in intercultural communication (A5) | Focuses on cultural identity and heritage, including perceptions of Chinese dance (B1), the reconstruction of Indigenous culture (B2), and the reconstruction of traditional dance teaching (B4) |
| Educational Reform and Teaching Methods | Discusses the necessity of specific teaching methods and educational reform, such as integrating recreational culture into pedagogy (A7) and the introduction of writing practices in cross-cultural dance (A11) | Emphasizes reform and inclusivity strategies within the education system, such as the professional development of physical education teachers (B8) and the impact of community art projects on disadvantaged children (B9) |
| Criticality and Reflection | Employs critical and reflective approaches, such as critical phenomenology (A2) and revealing cross-cultural significance through student performers' reflections (A10) | Not directly mentioned but embedded in critical reflection within education reform and professional development |
| Professional Development and Identity | Involves the professional development of teachers and educators' teaching experiences (A8) | Explicitly explores the identity and professional development of professional dancers, such as identity crises among dance practitioners (B3) and the development of professional identity through DMT (B14) |

**Table 10. Comparative Analysis of Core Issues—Differences**

| **Differentiation Category** | **Cross-Cultural** | **Indigenous Cultural** |
| --- | --- | --- |
| Geographical and Cultural Scope | Covers various countries and cultural backgrounds, focusing on cross-cultural exchange and educational reform within a globalized context | Primarily focuses on the culture and educational practices of a single country or region, with greater emphasis on local cultural identity and heritage |
| Research Perspective and Focus | Focuses on exploring cross-cultural exchange and the impact of globalization | Emphasizes local cultural identity, educational reform, and professional development, with attention to specific teaching practices and social value |
| Teaching Methods and Practices | Emphasizes diverse teaching methods and cross-cultural integration | Focuses on specific teaching experiences and local educational reform |

**Table 11. Core Perspectives in Intercultural Dance Education Research**

| **Research Perspective Category** | **Conceptual Implications** |
| --- | --- |
| Intercultural Exchange and Understanding | · Any form of isolation and segregation leads to mutual misunderstanding. Traditional songs and dances are suitable for fostering positive intercultural, religious, and ethnic exchanges and should be incorporated into teaching (A6). · The process of teaching and learning non-Western cultural dances plays a significant role in promoting intercultural learning in higher education (A10). · Practicing intercultural dance may be a way to enhance cultural understanding (A12). |
| Cultural  Adaptation and  Pedagogical Reform | · Pedagogical cultural adaptation is the process through which dance educators modify and negotiate teaching techniques and methods to suit new cultural environments and communities (A3). · Cultivating comprehensive cultural dance literacy, moving beyond a Eurocentric monocultural perspective (A8). · Introducing Dance/Movement Therapy (DMT) in non-Western cultural contexts requires adapting to students' learning styles while integrating local cultural perspectives on psychotherapy (A9). · When writing and dance practices are conducted in a conscious and interconnected manner, they can facilitate student learning in both areas (A11). |
| Race and Identity Formation | · Without acknowledging racialized discourse, dance curricula will continue to perpetuate narratives of racial inferiority, leading students to experience racialized "otherness" (A2). · Dance education for Indian classical dancers abroad must include improvisation training to enhance dance proficiency and foster the development of multi-group racial identity (A4). |
| Social Function of Dance Education | · Dance conveys social concepts such as community, authority, and respect (A1). · Soviet socialist body ideology influenced the Soviet-Canadian second-generation immigrant ballroom dancers and their teachers, shaping the direction of ballroom dancing in North America and Europe (A7). |
| Integration of Innovation and Tradition | · The protection of choreography must be pursued globally, with modern dance forms receiving equal emphasis as traditional forms (A13). · Dance arts education will continue to perpetuate both traditional and modern dance forms. Despite traditional practitioners questioning the authenticity of contemporary styles, their appeal makes them a vital entry point for new dancers (A13). |

*For the original references corresponding to the perspectives in intercultural research, please see Appendix F.

**Table 12. Core Perspectives in Indigenous Cultural Dance Education Research**

| **Research Perspective Category** | **Conceptual Implications** |
| --- | --- |
| Cultural Heritage and National Identity | · The role of Chinese dance in preserving cultural heritage in the digital age, particularly concerning collective and national identity (B1). · The positive impact of students acquiring additional cultural knowledge on learning specific ethnic dances, including the understanding of traditions, culture, and attitudes (B6). · Culturally-oriented dance has potential positive effects on the physical and mental health of professional dancers and communities, particularly regarding the health benefits of traditional West African dance (B12). |
| Pedagogical Methods and Practices | · Broadening mainstream pedagogical approaches by valuing the voices of elders and Indigenous Knowledge Systems (IKS), incorporating cultural practices such as dance into teaching methods, and emphasizing the value of lifelong learning (B2). · The philosophy of physical training should include creativity related to metacognitive qualities, such as self-reflection, self-efficacy, and resilience (B3). · Adjusting dance pedagogy through anthropological theories, emphasizing the necessity of creating open and trusting environments for successful teaching (B7). · Through the positive practice of DMT in the classroom, DMT educators can share experiences with the broader world of educational theory, promoting interdisciplinary practice (B14). · Community arts projects and equitable teaching frameworks are essential (B9).  · The application of anthropological theory allows dance pedagogy to be adapted according to the teacher's objectives and purposes (B8) |
| Cultural Reproduction and Creativity | · Dance education practices impact post-colonial countries' social justice pedagogies, aiding in the restoration of African cultural identity (B4). · The process of socialization may limit participants' creativity and agency, influencing the reproduction of dance culture and shaping individual and collective identities (B10). |
| Sociocultural Role of Dance | · Sekper, rooted in local animistic religious practices, has limited information available to outsiders (B5). · Frevo dance, as a dance of resistance, challenges the limitations of "tradition" and "authenticity," serving both as cultural heritage and a form of personal artistic expression (B11). · Dance plays a significant role in the development of multicultural education and culturally responsive pedagogy, helping children explore and express culture (B12). · Culturally-oriented dance has potential positive effects on the physical and mental health of professional dancers and students in higher education and community dance programs (B13). |

*For the original references corresponding to the perspectives in indigenous cultural research, please see Appendix G.

**Table 13. Comparative Analysis of Commonalities between Intercultural and Indigenous Cultural Perspectives**

| Commonality Category | Intercultural | Indigenous |
| --- | --- | --- |
| Cultural Exchange and Identity | Emphasizes the importance of intercultural exchange, viewing traditional dance as an effective medium for fostering intercultural understanding (A5, A9, A11) | Emphasizes that learning dance within specific cultural contexts deepens understanding and identification with that culture, aiding students in exploring and expressing themselves in a multicultural environment (B1, B6, B12) |
| Teaching Methods | Highlights the need for teachers to adapt their methods in intercultural contexts (A3, A7, A8, A10) | Stresses the importance of integrating indigenous knowledge systems and creating a trusting environment, focusing on the application of local culture and traditions in teaching (B2, B3, B7, B8, B9, B14) |
| Identity Formation | Addresses the negative impact of racialized discourse on students' identity, emphasizing the role of dance education in promoting multiethnic identity formation (A2, A4) | Focuses on the restoration of cultural identity in post-colonial nations, examining the impact of dance cultural reproduction on identity formation （B4, B10） |
| Social Function | Emphasizes the social functions of dance in intercultural contexts, such as the transmission of community authority and respect (A1, A6) | Focuses on the social roles of dance within specific cultures, including religious practices and expressions of resistance to tradition （B5, B11, B12, B13） |
| Integration of Innovation and Tradition | Advocates for the balanced preservation of both modern and traditional dance forms on a global scale (A12, A13) | Focuses on the impact of traditional dance on community and individual health, reflecting an emphasis on culturally-oriented approaches（B4, B10, B12, B13） |

**Table 14. Comparative Analysis of Differences between Intercultural and Indigenous Cultural Perspectives**

| **Difference Category** | **Intercultural** | **Indigenous** |
| --- | --- | --- |
| Cultural Exchange and Understanding | Focuses on intercultural adaptation and exchange | Emphasizes the protection of cultural heritage and national identity |
| Teaching Methods | Highlights cultural adaptation and integration within multicultural contexts | Focuses on broadening mainstream pedagogical approaches and valuing indigenous knowledge systems |
| Race and Identity Formation | Concerns with the impact of racialized discourse on students' identity | Examines the role of cultural reproduction in shaping individual and collective identity |
| Social Function | Focuses on the global social impact of dance | Concentrates on the social role of dance within local communities |
| Integration of Innovation and Tradition | Stresses the equal importance of modern and traditional dance | Focuses on the positive impact of culturally-oriented dance on community and individual health |

**Table 15. Methodological Strategies in Intercultural Dance Education**

| **Strategy Category** | **Conceptual Connotation** |
| --- | --- |
| Social Interaction and Cultural Transmission | · Employ social interaction, teaching, and performance practices to convey new forms of knowledge (A1)  · Use storytelling and music to activate specific movements, enhancing teacher-student relationships (A3)  · Create a safe space through humor to foster mutual sharing among students (A9) |
| Stratified and Group-Based Teaching | · Implement stratified group teaching to assign students to appropriate roles in dance productions (A1)  · Utilize methods such as group discussions and whole-class sharing (A9, A11) |
| Teacher Training and Trauma-Informed Dance Therapy | · Establish culturally relevant teacher training to help educators understand and convey cultural knowledge (A2)  · Employ trauma-informed dance therapy to aid students in healing from trauma and promote mental well-being (A2) |
| Authoritarian and Autocratic Teaching Methods | · Utilize authoritarian teaching methods, placing teachers in positions of power for rigorous training (A7) |
| Critical and Reflective Teaching | · Provide theoretical approaches to critical inquiry, emphasizing student independence and creativity (A8, A9) |
| Cultural Integration and Tradition | · Apply a unified teaching approach that integrates traditional costumes and props to reveal contextual knowledge of dance (A10)  · Reflect multiculturalism through the inclusion of Flamenco culture, West African dance, and more (A12)  · Incorporate local culture through practices such as “Tai Chi” and “meditation” (A9)  · Teachers use Eastern Indian folk dance to shape students' ideologies (A5) |
| Inclusivity and Equity | · Establish scholarships to support student learning and development, with a focus on diversity and inclusivity in curriculum design (A1) |

*Note: A4, A6, and A13 do not provide descriptions of methodological strategies. The original coding information for intercultural strategies can be found in Appendix H.

**Table 16. Methodological Strategies in Indigenous Cultural Dance Education**

| **Strategy Category** | **Conceptual Connotation** |
| --- | --- |
| Cultural Collaboration and Exchange | ·Emphasizes cultural collaboration and experiential exchange in teaching strategies, promoting cultural transmission and understanding through concerts, dance classes, special events (B1), carnivals, and other activities (B11) |
| Inclusivity and Collaboration | ·The teaching environment emphasizes inclusivity and collaboration, utilizing indigenous methods such as Yarning to encourage participants to share and learn in an inclusive, collaborative setting (B2)  ·Mixed teaching and cooperation with peers and families emphasize collaboration over competition, reflecting inclusivity and collaboration (B9 |
| Cultural Responsiveness and Critical Reflection | ·Teaching methods focus on cultural responsiveness, developing curriculum content and pedagogical knowledge through interaction, problem-solving, observation, feedback, and critical reflection (B12) |
| Care and Support | ·Teachers' care and support for students are reflected in the teaching process, emphasizing the combination of effort and caution through posture adjustments and attention to students' physical needs (B9) |
| Practice and Performance | ·Emphasizes motivating students through competition and performance, enhancing their skills and confidence (B  6，B10, B11) |

*Note: B3, B4, B5, B7, B8, B13, and B14 do not provide descriptions of methodological strategies. The original coding information for indigenous cultural strategies can be found in Appendix I.

**Table 17. Comparative Analysis of Commonalities in Methodological Strategies between Intercultural and Indigenous Cultural Perspectives**

| **Commonality Category** | **Intercultural** | **Indigenous** |
| --- | --- | --- |
| Cultural Collaboration and Exchange | Promotes cultural transmission and understanding through social interaction, storytelling, music, and multicultural forms such as Flamenco and West African dance (A1, A3, A12) | Advances cultural dissemination and exchange through activities such as concerts, dance classes, and carnivals (B1, B11) |
| Inclusivity and Collaboration | Fosters inclusivity and equity through diverse teaching methods, including stratified teaching and group discussions (A1, A9) | Uses indigenous methods like Yarning and mixed teaching to encourage collaboration over competition, with a focus on inclusivity in education (B2, B9) |
| Critical Reflection | Emphasizes student independence and creativity through critical inquiry and reflective teaching (A8, A9) | Develops curriculum content and pedagogical knowledge through interaction, problem-solving, feedback, and critical reflection (B12) |
| Care and Support | Promotes students' mental health through trauma-informed dance therapy and supportive teaching practices (A2) | Stresses the importance of teachers' care for students' physical and psychological well-being, supporting their overall development (B9) |
| Practice and Performance | Integrates performance activities and multiculturalism into education (A1, A10, A12) | Cultivates students' practical skills and confidence through competitions and performances (B6，B10, B11) |

**Table 18. Comparative Analysis of Differences in Methodological Strategies between Intercultural and Indigenous Cultural Perspectives**

| **Difference Category** | **Intercultural** | **Indigenous** |
| --- | --- | --- |
| Cultural Integration and Collaborative Exchange | Emphasizes cultural integration and tradition through unified teaching methods, combining traditional costumes and props , and the integration of different cultures, such as the ideological shaping of Indian folk dance | Focuses on the preservation and display of local culture, showcasing cultural characteristics through activities like carnivals |
| Teacher Training and Trauma-Informed Dance Therapy | Includes culturally relevant teacher training and trauma-informed dance therapy design | / |
| Authoritarian and Autocratic Teaching Methods | The application of authoritarian and autocratic teaching methods places teachers in positions of authority for rigorous training | / |
| Social Interaction and Cultural Transmission | Emphasizes social interaction and interaction strategies that convey new knowledge through teaching and performance practices | Focuses on promoting understanding and identification through cultural collaboration and exchange |

**Table 19. Research Conclusions in Intercultural Dance Education**

| **Research Conclusion Category** | **Conceptual Content** |
| --- | --- |
| Cultural Adaptation and Identity | · Cultural Adaptation: Enhancing students' adaptability in cross-cultural environments by adjusting teaching strategies and integrating diverse cultural elements (A3, A7, A10). · Cultural Identity: Fostering students' sense of cultural identity through dance education, while promoting understanding and respect for other cultures, thereby strengthening cultural identity (A3, A5, A6). |
| Diverse Teaching Methods | · Multifaceted Approaches: Promoting comprehensive student development by integrating language and music, modifying movements, modern choreography, and improvisation to innovate cultural works (A3, A4, A11, A13). · Integrated Teaching: Combining dance with other disciplines such as practical theology and figurative writing to enhance students' overall abilities and interdisciplinary thinking (A1, A5). |
| Teacher Roles and Capabilities | · Teacher's Role: Teachers serve not only as knowledge transmitters but also as cultural mediators and facilitators of adaptability in the classroom (A12). · Professional Development: Teachers need to actively address students' racial differences and develop new professional identities to adapt to a multicultural context (A2, A9). |
| Cultural Inclusivity and Equity | · EEP Dance Teaching Model: Overcoming traditional cultural biases by enhancing cultural inclusion and educational equity (A8). |
| Student Agency | · Learning Agency: Through reflection and inquiry, students actively construct cross-cultural meaning, enhancing their cultural understanding and learning experience (A10). · Learning Experience: Through diverse learning approaches, students deepened their understanding and mastery of dance and its underlying cultural connotations (A1, A4). |

*Note: The original coding information for the intercultural research conclusions can be found in Appendix J.

**Table 20. Research Conclusions in Indigenous Cultural Dance Education**

| **Research Conclusion Category** | **Conceptual Content** |
| --- | --- |
| Cultural Transmission and Identity | · Complexity of Chinese Dance Culture: Students’ insufficient understanding of Chinese dance culture may hinder the global integration of Chinese culture (B1, B7). · Transmission of Indigenous Knowledge: Jagun's indigenous knowledge, passed down by elders to the younger generation, promotes cultural identity and strengthens community ties (B2). · The Role of Ethnic Dance in Greek Education: Ethnic dance promotes cultural homogeneity and national identity (B6). · Cultural Significance of Ballet Folklórico: Assists Mexican-American students in developing identity and pride (B9). |
| Teaching Methods | · Limitations of Western Pedagogies: Western-centric pedagogies reinforce implicit norms and stereotypes, suggesting the need for self-reflection and resilience training (B3). · Teaching Methods of Guerreiros do Passo: Preserves tradition while encouraging student innovation, fostering cultural debate (B11). · Culturally Responsive Strategies in Dance Teaching: Strengthens connections between teachers and students, among students, and between students and dance (B12). · Embodied Self-Reflection in DMT Students: Enhances awareness of cultural, professional, and bodily selves through experiential and embodied learning (B14). |
| Teacher Capabilities | · Inadequate Teacher Capabilities: Teachers’ limited ability to demonstrate and execute traditional dance calls for improvements in teacher training and pedagogical methods (B4, B5). · Challenges for Japanese Physical Education Teachers: Japanese PE teachers face challenges in creating broad-based instructional plans that respect the regional characteristics of traditional dance and address inappropriate teaching formats (B8). |
| Social Function and Impact | · Impact of Dance Competitions: Intensive training for dance competitions helps shape individual traits, reproduce cultural preferences, and build team spirit among participants (B10). · Impact of West African Dance: West African dance contributes to dancers' physical, psychological, and social well-being, connecting culture and community, although time and financial constraints pose barriers to participation (B13). |

*No research conclusion is provided for B7; the original coding information for the indigenous cultural research conclusions can be found in Appendix K.

**Table 21. Comparative Analysis of Commonalities in Cross-Cultural vs. Indigenous Cultural Research Conclusions**

| **Commonality Category** | **Cross-Cultural** | **Indigenous Culture** |
| --- | --- | --- |
| Cultural Transmission and Identity | Global Adaptation: Utilizing diverse cultural elements to help students adapt to a global environment (A3, A7, A10)  Cross-Cultural Understanding: Enhancing recognition and respect for one's own and others' cultures (A3, A5, A6) | Indigenous Transmission: Emphasizing the cultural role of indigenous knowledge and folk dance (B2, B6,)  Cultural Identity: Promoting identification with one's own culture (B1, B7, B9) |
| Diverse Teaching Methods | Integrated Approaches: Using various methods and interdisciplinary integration to promote development (A3, A4, A11, A13)  Interdisciplinary: Integrating multiple fields to enhance overall abilities (A1, A5) | Strategic Response: Focusing on specific teaching strategies and cultural responsiveness (B3, B11)  Teaching Adaptation: Addressing cultural differences through teaching strategies (B12, B14) |
| Teacher Roles and Competencies | Multiple Roles: Teachers as transmitters of knowledge and facilitators of cultural adaptation (A2, A9, A12)  Professional Development: Emphasizing adaptation to cultural differences (A2, A9) | Skills Challenges: Discussing teacher training and skill enhancement (B4, B5)  Regional Adaptation: Adjusting teaching methods to accommodate regional cultural characteristics (B9) |

**Table 22. Comparative Analysis of Differences in Cross-Cultural vs. Indigenous Cultural Research Conclusions**

| **Difference Category** | **Cross-Cultural** | **Indigenous Culture** |
| --- | --- | --- |
| Cultural Scope of Teaching Content | Involves multicultural integration and a global perspective | Focuses on cultural transmission specific to certain countries or regions |
| Social Purpose of Dance Education | Emphasizes cross-cultural understanding and identity formation | Discusses the role of dance education in social and political spheres |
| Practical Innovations in Dance Education | Encourages free improvisation and interdisciplinary dance teaching to foster student creativity and understanding | Emphasizes implementing cultural response strategies and experiential learning in dance education to strengthen student connections with dance |
